# Early changes in bone mineral density measured by digital X-ray radiogrammetry predict up to 20 years radiological outcome in rheumatoid arthritis

**DOI:** 10.1186/ar3259

**Published:** 2011-02-23

**Authors:** Meliha C Kapetanovic, Elisabet Lindqvist, Jakob Algulin, Kjell Jonsson, Tore Saxne, Kerstin Eberhardt, Pierre Geborek

**Affiliations:** 1 Department of Clinical Sciences, Lund, Section of Rheumatology, Lund University, Kioskgatan 3, Lund SE-221 85, Sweden; 2 Sectra AB, Teknikringen 20, Linköping, SE-583 30, Sweden; 3 Department of Clinical Sciences, Lund, Center for Imaging and Physiology, Lund University, Getingevägen 4, Lund, SE-221 85, Sweden

## Abstract

**Introduction:**

Changes in bone mineral density (BMD) in the hand as evaluated by digital X-ray radiogrammetry (DXR) of the second to fourth metacarpal bones has been suggested to predict future joint damage in patients with rheumatoid arthritis (RA). This study's objective was to investigate whether DXR-BMD loss early in the course of the disease predicts the development of joint damage in RA patients followed for up to 20 years.

**Methods:**

A total of 183 patients (115 women and 68 men) with early RA (mean disease duration, 11 months) included from 1985 to 1989 were followed prospectively (the Lund early RA cohort). Clinical and functional measures were assessed yearly. Joint damage was evaluated according to the Larsen score on radiographs of the hands and feet obtained in years 0 to 5 and years 10, 15 and 20. These radiographs were digitized, and BMD of the second to fourth metacarpal bones was evaluated by DXR. Early DXR-BMD change rate (that is, bone loss) per year calculated from the first two radiographs obtained on average 9 months apart (SD ± 4.8) were available for 135 patients. Mean values of the right and left hand were used.

**Results:**

Mean early DXR-BMD loss during the first year calculated was -0.023 g/cm^2 ^(SD ± 0.025). Patients with marked bone loss, that is, early DXR-BMD loss above the median for the group, had significantly worse progression of joint damage at all examinations during the 20-year period.

**Conclusions:**

Early DXR-BMD progression rate predicted the development of joint damage evaluated according to Larsen score at year 1 and for up to 20 years in this cohort of early RA patients.

## Introduction

Rheumatoid arthritis (RA) is an inflammatory disease characterized by chronic synovial inflammation commonly associated with destruction in cartilage and bone tissue. Changes in bone metabolism during the course of RA are usually divided into periarticular bone loss with or without focal articular bone erosion and generalized osteopenia manifested by loss of both trabecular and cortical bone. Periarticular bone loss occurs early and often before erosion is apparent. This is linked to the inflammatory process and may be driven by locally released inflammatory mediators such as the receptor activator of nuclear factor (NF)-κB and its ligand RANKL and the decoy receptor of RANKL, osteoprotegerin. These findings have been extensively investigated and reviewed [1-9]. In RA, periarticular bone loss is seen predominantly in the hands and feet, which are affected early in the disease course. Thus, bone loss may be a marker of disease activity, and it has been suggested that it may also relate to future joint damage [4,5,8].

Early loss of bone mineral density (BMD) in the hand has been shown to covariate with radiographic joint damage as measured by the Sharp-van der Heide (SvdH) or Larsen score in RA patients [4-11]. However, outcome measures do not necessarily agree. The two more established radiological scores in RA, the Larsen and the SvdH, demonstrate only a modest correlation. This may partly be explained by the fact that the Larsen score is more global, while the SvdH score measures specific joint regions [12-14].

The bone mineral density measured by digital X-ray radiogrammetry (DXR-BMD) determination has the advantage of being well standardized and not subject to the interpretation and measurement errors that are inherent in both the Larsen and the SvdH scoring systems. Furthermore, DXR-BMD is reliable in quantifying demineralization and/or osteoporosis, which have been shown to be only imprecisely verified with conventional radiography. The radiographic scoring methods and DXR-BMD can be performed on historical radiographs, provided that these are of sufficient quality and taken in a standardized fashion. This is in contrast to other methods of BMD measurements such as ultrasound, dual-emission X-ray absorptiometry and peripheral quantitative computed tomography, all of which have as a focus the measurement of BMD, are not retrospectively applicable to standard radiographs of the hands and sometimes focus on generalized osteopenia [6-8,15-18].

In Lund, Sweden, we have prospectively monitored a cohort of early RA patients since 1985 and have more than 20 years of follow-up information on radiographic and clinical outcomes [19,20]. In the present study, we have examined the relationship between early DXR-BMD changes and short- and long-term outcomes as measured radiographically by the Larsen score of the hands and feet. Furthermore, we have examined the relationship between baseline BMD, Larsen score and early Larsen progression and long-term radiographic outcome.

## Materials and methods

### Patients

A total of 183 patients (115 women and 68 men) with early definite RA who had a mean symptom duration of 11 months (SD ± 7; range, 0 to 24 months) included between 1985 and 1989 were followed prospectively (the Lund early RA cohort). Clinical and functional measures were assessed, as previously reported, at least once yearly [19]. The validated Swedish version of the Health Assessment Questionnaire (HAQ), which includes the use of aids, was used [21]. Approval from the Ethical Review Board at Lund University (LU 525-02) and informed consent from each patient were obtained for this study. Throughout the study all patients with active disease were offered treatment with disease-modifying antirheumatic drugs (DMARDs) according to current clinical practice. About 50% of patients from the original cohort started treatment with DMARDs within 1 year after diagnosis. D-penicillamine and antimalarial drugs were most commonly used in the early years, and methotrexate became most frequently used during the 1990s.

In total, 48 patients from the original cohort were excluded from the analysis. In three cases, only hand and no feet radiographs were available as the first radiographs; in eight cases, the first DXR measurements were made on prediagnosis inclusion radiographs which were not Larsen scored; and in an additional 37 patients, radiographs were taken using a magnifying analogous technique precluding DXR measurements. There were no statistically significant differences regarding patient characteristics between included and excluded patients.

DXR was developed as a computerized method of radiogrammetry to measure cortical bone thickness in diaphysis of the second to fourth metacarpal bones using standard hand radiographs. Thus, DXR-BMD quantifies only the cortical bone tissue where the bone metabolism is minor compared to trabecular bone tissue [9]. The method has been described in more detail elsewhere [9,15-17]. Briefly, the early DXR-BMD change rate (bone loss) per year, expressed in grams per square centimeter, was calculated on the basis of the first two available conventional radiographs of the hands. Exact dates of the radiographs were used to calculate the yearly change rate. The same radiographs were used for both radiographic scoring and measurement of hand bone density. The radiographs were digitized to 300 dpi, 12-bit gray scale format with DICOM software using a Vidar Diagnostic Pro Plus digitizer (VIDAR Systems Corp., Herndon, VA, USA), and the resulting digital radiographs were analyzed using dxr-online software (Sectra, Linköping, Sweden). The dxr-online software recognizes regions of interest around the narrowest part of the second, third and fourth metacarpal bone diaphysis and automatically measures the BMD through a combination of radiogrammetry and textural analysis. Mean values of the right and left hand were used. The smallest detectable difference in elevated early DXR-BMD loss has been shown to be 0.0048 g/cm^2 ^[7].

### Larsen score

Joint damage was evaluated on radiographs of the hands and feet according to the Larsen method as described previously [13]. In short, 32 joints in the hands and feet were evaluated. Each joint was compared to a standard reference film, and changes were graded from 0 to 5, with 0 being normal; 1 being joint space narrowing, soft tissue swelling or periarticular osteoporosis; and 2 to 5 representing a progressively increasing degree of erosion and destruction. A joint damage score was calculated by adding all scores, with the scores for joints in the wrists multiplied by 5, resulting in a range of 0 to 200. Radiographs were obtained at inclusion in the study as well as after 1, 2, 3, 4, 5, 10, 15 and 20 years. Early progression of Larsen scores per year was measured as the difference between the first two available sets of both hand and feet radiograms using the exact dates of the radiograms to calculate the progression rate per year (Larsen units/year).

### Statistics

Early DXR-BMD changes stratified according to the median and upper and lower halves were compared to each other. No imputation of missing data was performed. Differences between the groups were calculated using the χ^2 ^test for ordinal variables and the Mann-Whitney *U *test for numerical variables. Influences of age and sex on baseline BMD, early DXR-BMD change and baseline Larsen score were estimated by performing analysis of covariance (ANCOVA). To maximize statistical power, differences between Larsen scores at 10 years were analyzed using a generalized linear regression model adjusted for age, sex and baseline values of the variable tested. This was done because 69 patients died during follow-up (the majority after 10 years), and thus only contributed information for a more limited follow-up period. Correlations were calculated using Spearman's rank correlation coefficient. Values are given as means with 95% confidence intervals (95% CIs) unless stated otherwise. A *P *value < 0.05 was considered statistically significant.

## Results

### Patients and characteristics according to early BMD-DXR

Complete data were available for 135 patients. Mean (± SD) DXR-BMD at baseline was 0.593 ± 0.08 g/cm^2^. Mean (± SD) DXR-BMD progression rate (bone loss), defined as change in DXR-BMD between the first two existing radiographs was -0.023 g/cm^2 ^± 0.025.

Patient characteristics stratified according to the median value (-0.019) of early DXR-BMD loss are presented in Table 1. This stratification identified several differences in baseline variables. Patients with high bone loss were older, had longer symptom duration at diagnosis and had higher erythrocyte sedimentation rate (ESR), C-reactive protein (CRP), HAQ scores and Disease Activity Score using 44 joint counts (DAS44), while sex and Larsen score were not significantly different. ANCOVA showed that older patients had both significantly lower baseline BMD (*P *< 0.001) and higher early DXR-BMD changes (*P *< 0.001), while females only had lower baseline BMD (*P *< 0.001), but not significantly higher early DXR-BMD changes (*P *= 0.690).

**Table 1:** Patient characteristics at diagnosis stratified according to the median of the early DXR-BMD loss^a^

	High bone loss	Low bone loss	
	(higher than median)	(less than median)	*P *value
Patient characteristics	(*n *= 69)	(*n *= 66)	
Baseline characteristics			
Mean age, yr (± SD)	57.7 (11.4)	46.4 (11.4)	<0.001
Mean symptom duration at inclusion, months (± SD)	10.6 (6.8)	13.0 (7.1)	0.035
Mean HAQ, 0 to 3 (± SD)	1.0 (0.6)	0.7 (0.6)	0.004
Mean DAS44, 0 to 10 (± SD)	3.5 (1.1)	3.0 (1.0)	0.024
Mean CRP, mg/l (± SD)	34 (36)	18 (31)	<0.001
Mean ESR, mm/1 hour (± SD)	46 (30)	28 (25)	<0.001
Female, %	62%	67%	ns
RF-positive, %	81%	85%	ns
ACPA-positive, %	78%	74%	ns
Smoker, %	34%	32%	ns
Mean Larsen score, 0 to 200 (± SD)	10.2 (9.4)	6.6 (6.2)	0.100
Mean BMD, g/cm^2 ^(± SD)	0.57 (0.09)	0.62 (0.07)	<0.001
Early DXR-BMD loss, g/cm^2 ^(± SD)	-0.0418 (0.02)	-0.0034 (0.008)	<0.001
Early DXR-BMD loss, %	-7.6	-0.57	<0.001
Treatment during total observation period, %			
DMARDs ever, %	80%	71%	ns
MTX ever, %	39%	42%	ns
Prednisolone ever, %	48%	47%	ns

^a^SD, standard deviation; DXR-BMD, bone mineral density (BMD) measured by digital X-ray radiogrammetry; HAQ, Health Assessment Questionnaire; DAS44, Disease Activity Score using 44 joint counts; ESR, erythrocyte sedimentation rate; CRP, C-reactive protein; RF, rheumatoid factor; ACPA, anticitrullinated peptide antibodies; DMARDs, disease-modifying antirheumatic drugs; MTX, methotrexate; ns, not significant. Medication refers to treatment for the whole observation period.

### Early DXR-BMD association with and prediction of Larsen score

On the basis of univariate linear regression analysis, early bone loss was significantly associated with higher Larsen score at year 10 as were ESR and CRP levels, HAQ score and Larsen score at baseline. ANCOVA showed that the adjusted 10-year Larsen score in the group with high early bone loss (mean, 95% CI) was 30.2 (95% CI, 15.7 to 44.7) above the group with low early bone loss (*P *< 0.001).

In multivariate regression analysis, high early DXR-BMD loss (patients with early bone loss levels above the median), baseline Larsen scores and ESR levels remained predictive for the Larsen score at year 10 (Table 2). In patients with erosive disease at baseline (that is, Larsen score at baseline ≥2) who had early DXR-BMD, the median value was still associated with a higher Larsen score at 10-year follow-up (*P *= 0.031). Very few patients (*n *= 2) in this cohort had already been treated with prednisolone at RA diagnosis (baseline), and therefore this variable was not entered into the regression model.

**Table 2:** Impact of high early DXR-BMD loss and baseline demographic and disease characteristics on Larsen score at year 10 (linear regression model)^a^

Patient demographics and disease characteristics	B	*P_*value
Early bone loss above median (yes/no)	16.572	0.037
Sex	3.573	0.620
Age	-0.462	0.134
ESR	0.540	0.000
Larsen score at baseline, 0 to 200	0.807	0.068
ACPA (yes or no)	-1.371	0.862

^a ^B, the estimated regression coefficient; ESR, erythrocyte sedimentation rate; ACPA, anticitrullinated peptide antibodies; high early DXR-BMD loss is defined as bone loss above median value.

**Figure 1:**
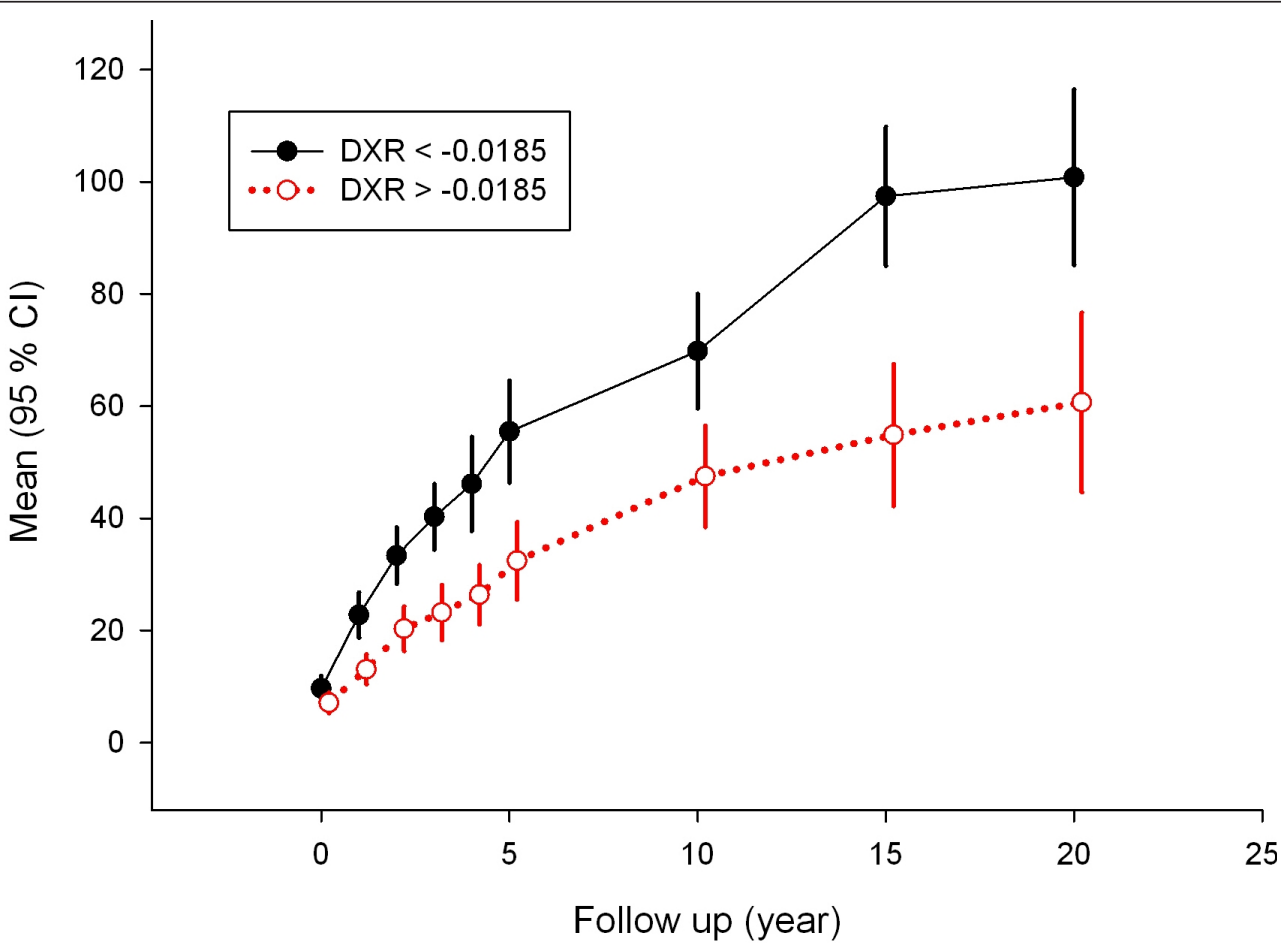
**Radiological Larsen score progression over time after stratification according to the median (-0.0185 g/cm**^**2 **^**per year) of early bone mineral density measured by digital X-ray radiogrammetry (DXR-BMD) change**. High loss (black solid line) versus low loss (red dotted line) of DXR-BMD. Values are given as means with 95% confidence intervals (95% CIs). The larger 95% CIs at 15 and 20 years illustrate the decreasing number of patients at these time points.

Larsen scores at years 0, 1, 3, 4, 5, 10, 15 and 20 stratified according to the median of early DXR-BMD loss are given in Figure 1. There is a diverging trend during the whole follow-up period, which is most marked in the first 5 years. The increase in 95% CI at the later time points illustrates the increasing number of deceased patients. When using the smallest detectable difference, 0.0048 g/cm^2^/year [7], as a cutoff for elevated early DXR-BMD loss, a total of 97 patients (72%) were in the higher bone loss group, while 38 patients (28%) were in the lower bone loss group. Plotting the patients with early bone loss >0.0048 compared to those with lower early bone loss yielded results similar to those of median stratification (data not shown). During the first 5 years, the Larsen score deteriorated at a faster pace in patients with elevated early bone loss. However at time points 10, 15 and 20 years, these differences were not significant between the groups. The Spearman correlations between early bone loss and Larsen scores at years 1, 2, 3, 4, 5, 10 and 20 were all significant, with Spearman's ρ values between 0.359 and 0.223.

### Comparing DXR-BMD for Larsen scores for hands and feet

When looking at Larsen scores for hands and feet separately, the clear differences between groups stratified according to early DXR-BMD loss remained for the hands. For the Larsen scores of the feet, the picture was similar, with the group with high early DXR-BMD loss always having higher mean Larsen scores, but at most time points the differences were nonsignificant (data not shown).

### DXR-BMD progression rate over time

To study the progression rate of DXR-BMD loss over time, we calculated the data for the yearly progression rates between time points 0 and 1, 2, 3 and 4 years, which were (means and 95% CIs): 2.2 (1.3 to 3.0), 2.2 (1.5 to 2.9), 2.7 (2.0 to 3.3) and 2.3 (1.8 to 2.8), respectively. There was an overall trend of decreasing DXR-BMD progression rate over time, but the limited number of patients precludes firm statistical confirmation.

### Association with and prediction of early Larsen changes on later Larsen scores

**Figure 2:**
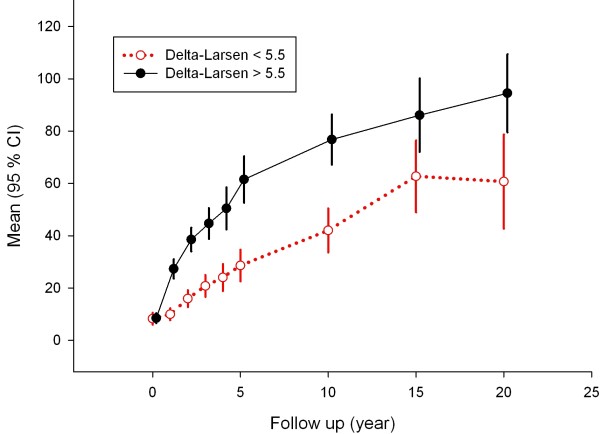
**Radiological Larsen score progression over time after stratification according to the median (5.5 units) of early change in Larsen score**. High (black solid line) versus low (red dotted line) early ΔLarsen scores. Values are given as means with 95% confidence intervals (95% CIs). The larger 95% CIs at 15 and 20 years illustrate the decreasing number of patients at these time points.

After stratifying for the median of early changes in Larsen scores, a very similar picture to that observed for early DXR-BMD changes was found (Figure 2). Also, here there is a diverging trend during the whole follow-up period, and ANCOVA showed that the adjusted 10-year Larsen scores (95% CI) in the group with more elevated early Larsen score progression was 32.3 (20.6 to 44.0) above the group with low early Larsen score progression. The association between early Larsen score changes with 10-year Larsen scores was 0.540 (Spearman's ρ).

### Baseline BMD or Larsen scores and late Larsen scores

**Figure 3:**
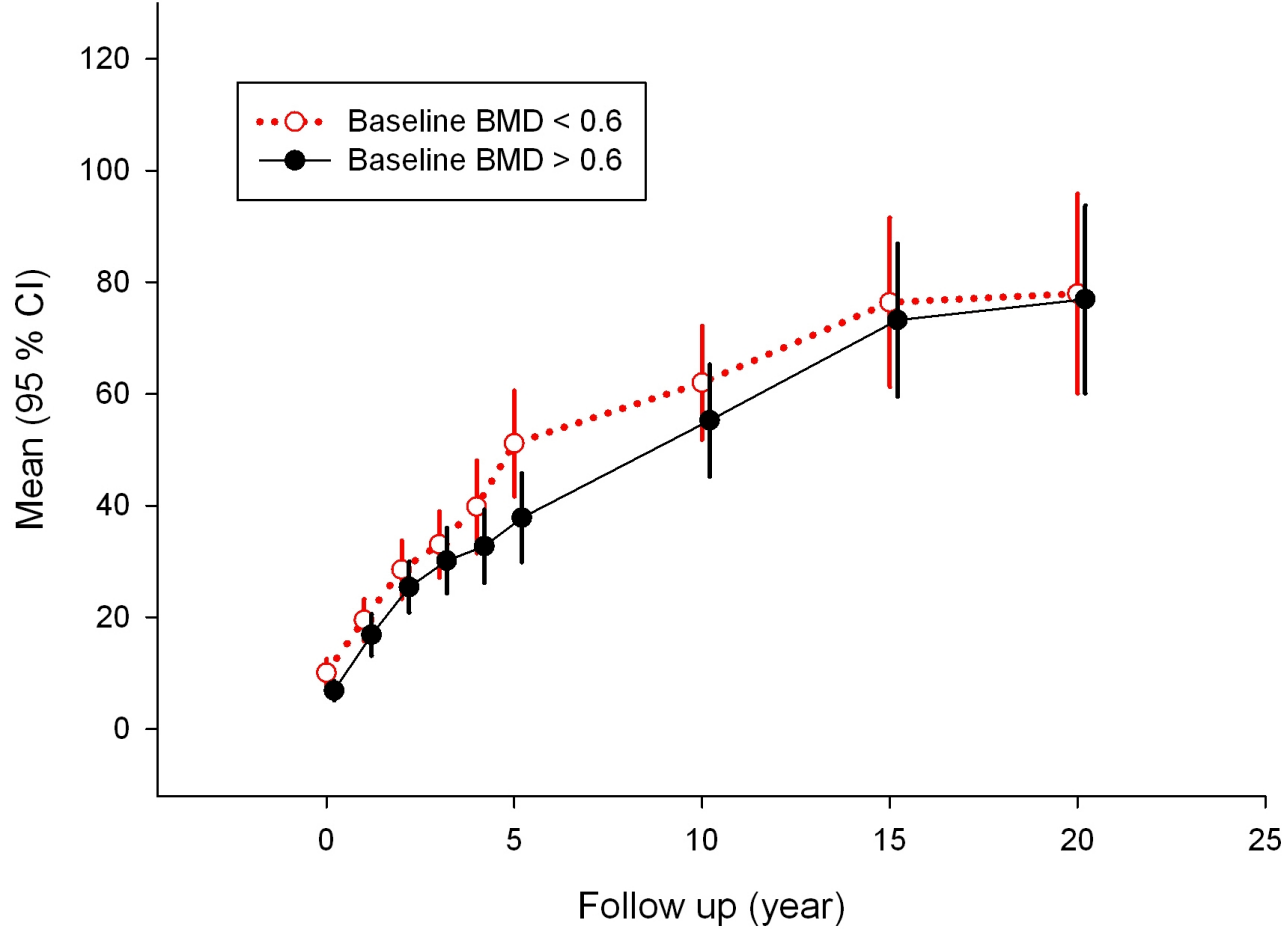
**Radiological Larsen score progression over time after stratification according to median (0.6 g/cm**^**2**^**) baseline BMD**. High BMD (black solid line) versus low BMD (red dotted line) values at baseline are shown.

**Figure 4:**
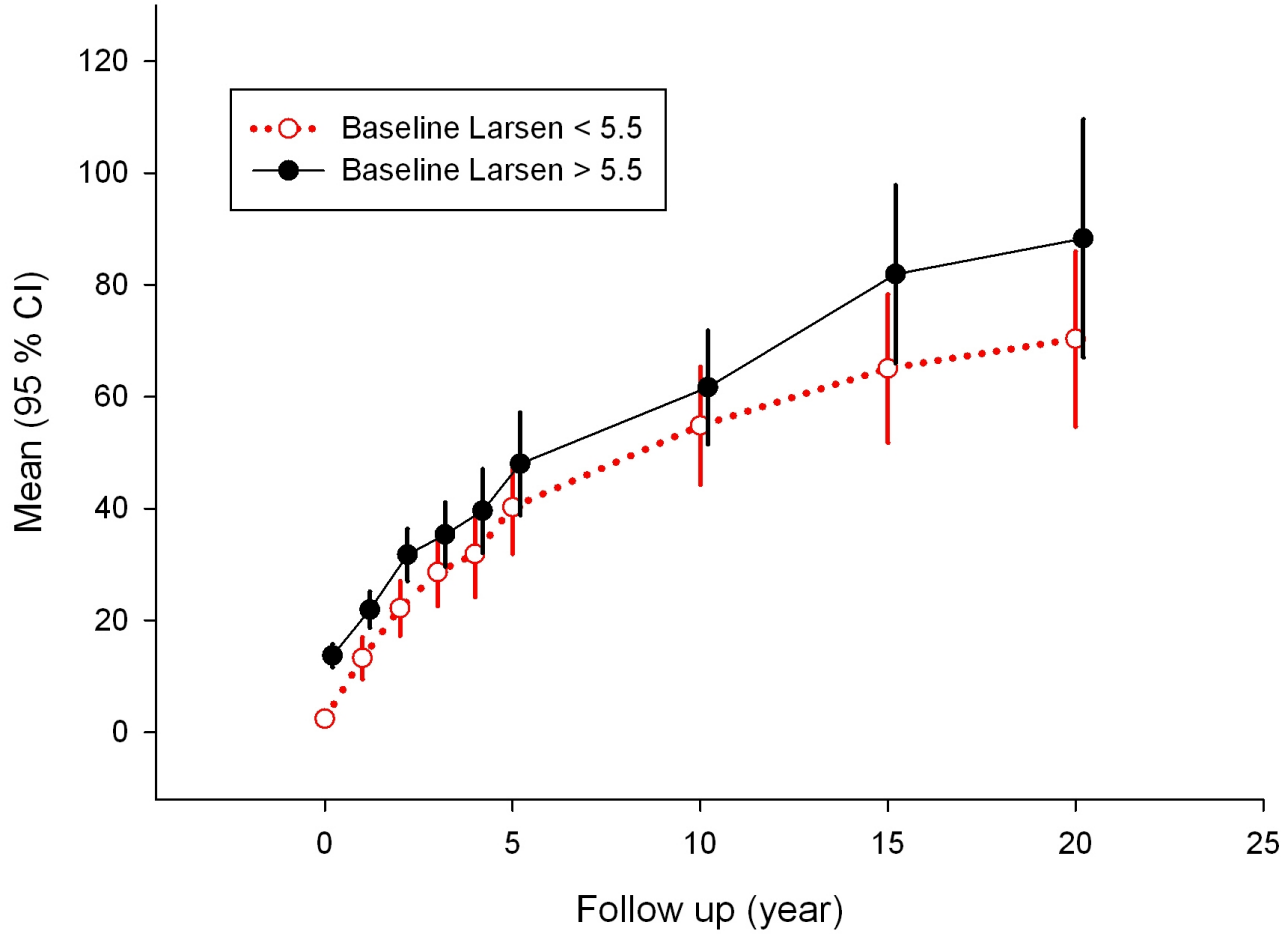
**Radiological Larsen score progression over time after stratification according to median (5.5 units) of baseline Larsen score**. High Larsen score (black solid line) versus low Larsen score (red dotted line) at baseline is shown.

Median stratification of baseline BMD or Larsen score did not identify any trends of predictability for long-term radiological progression as measured by the Larsen score (Figures 3 and 4). The Spearman's ρ correlation between 10-year Larsen score and baseline BMD was -0.056, and between 10-year Larsen score and baseline Larsen score it was 0.199.

## Discussion

In the present report, we have demonstrated that early bone mineral loss in the hands of patients with RA predict joint damage as measured by the radiographic Larsen score over a 20-year observation period. The difference was seen as early as 1 year after baseline all the way to 20-year follow-up.

We could not verify that DXR-BMD loss is more marked in the early phase of the disease, which has been reported by others, but our study had more limited power [4-11]. Overall our results are consistent with those of other studies. A longitudinal observational study including early RA patients with disease duration <1 year showed that changes in DXR-BMD of the second to fourth metacarpal bones at year 1 were specific and sensitive in identifying both patients who developed erosions scored using both the Larsen and SvdH methods and those whose existing erosion had progressed at year 4 [4]. Similarly, in RA patients with disease duration up to 4 years, hand bone loss measured by DXR-BMD at year 1 was predictive of subsequent radiographic damage scored by the SvdH method at 5 and 10 years [7].

We did not attempt to study the implication of late DXR-BMD loss in this study, since we aimed to find early predictors of outcome. On the other hand, the clinical value of early bone mineral loss as a predictor of future joint damage as measured by Larsen score should not be overemphasized. The relationship is well validated on the group level, but cannot be recommended for use as the only predictor as illustrated by the relatively low correlations. On the other hand, early progression in Larsen score also shows a clear predictive value for later joint damage, but the low correlations revealed that this information also must be confined to the group level. Early bone loss is a risk factor, in addition to other known risk factors. Thus, although statistically significant predictive value for RA prognosis can be found for several early findings such as the presence of rheumatoid factor, anticyclic citrullinated peptide antibody, elevated ESR level and cartilage oligomeric matrix protein level, their value in clinical decisions regarding treatment of the individual patient must not be overemphasized [22-29]. Treatment decisions rely heavily on several aspects not covered by conventional predictive analyses, such as perceived pain, working situation, deteriorating function and so on.

The findings that early DXR-BMD loss in the cortical shaft of metacarpal bones is predictive of later progression of joint-related hand Larsen score was not unexpected because of the close vicinity of these structures. We were not able to demonstrate an unequivocal relationship between early DXR-BMD loss in the metacarpal bones and Larsen score in the foot, even though there was a quite clear trend for such a relationship as has been reported for SvdH [11]. However, in the Larsen scoring system, as in the SvdH scoring system, the feet do not have the same number of joints as the hands, thus giving a ceiling effect with reduced possibility of finding differences [12-14]. This could at least partly explain the nonsignificant differences found in the Larsen scores of the feet. Methods of using DXR-BMD have not been developed for the metatarsal bones, thus precluding direct comparisons of hand and foot bone loss as well as a possible relationship between more generalized bone loss and multiple joint damage as measured by the total Larsen score.

Several factors are known to promote osteopenia, such as female sex, older age and severe RA [30,31]. It was therefore not unexpected to find that the degree of early DXR-BMD loss identified patients with different baseline characteristics. Thus early markers of more severe disease, such as high HAQ score, high DAS44 score and high ESR and CRP levels, were all more common among the patients with higher DXR-BMD loss. Also older age contributed to high early bone loss, while rheumatoid factor status or anticitrullinated peptide antibody positivity and sex did not. The low numbers of patients taking systemic glucocorticoids represent the therapeutic tradition in the 1980s and prevent any meaningful analysis of this cause of osteopenia.

The strength of the DXR-BMD technique is that it is well standardized, can be used to perform a large number of objective measurements and can be done on existing radiographs. However, the radiographs must be taken in a standardized fashion if the results are to be given in grams per square centimeter. DXR-BMD can be somewhat more generalizable if the results are given as percentages, which are sufficient to measure relative changes. Both the Larsen and SvdH scores require experienced and licensed readers and still have a sizable reading error [12], a shortcoming that DXR-BMD has minimized, although the radiographic equipment used can influence the results to a minor degree. DXR-BMD is able to quantify only cortical and not trabecular bone mass, thus limiting its use as a general osteopenia measure. However, periarticular cortical bone loss, in some cases measured by DXR-BMD, has been shown to predict erosive disease in RA [4-11].

The strengths of the current study include its community-based recruitment, its prospective gathering of a large amount of laboratory and clinical information and its almost complete follow-up. The exclusion of the sizable proportion of RA patients who died during follow-up could be argued for. However, they did represent a group of RA patients that presumably had several severity markers in common, and the exclusion of these patients could seriously hamper the results of the study. We also refrained from extrapolating and imputing missing Larsen scores, since this also leads to several assumptions and limitations in this restricted sample size.

The limitations of the present study include the retrospective approach of DXR-BMD measurements on preexisting radiographs. This accounted for the loss of 48 patients. However, their baseline characteristics were largely similar to those of the total group of patients. Also, the total sample size was not very large, but this was one of the very first early RA cohorts recruited, and it represents what one center could bear during a total of >25 years of follow-up. Thus, although some of the finer details may not always be statistically verified, the cohort size permits firm conclusions on several robust sets of data such as those in the present report. It must be remembered that in comparing data with more recent early RA studies, in which new and often more liberal criteria sets are being used, we used the 1958 American Rheumatism Association criteria to establish definite diagnoses of RA in the present study [32]. Also, the introduction of new and potentially bone loss-protective treatment cohorts must be accounted for in more recent early RA studies when comparing them with the Lund early RA cohort treated according to the clinical standards used over 25 years ago.

## Conclusions

Early DXR-BMD loss in the present study predicted future joint damage as measured by Larsen score both in the short-term perspective (1 year), which confirms previous studies that used the SvdH score, and for the first time in the very long-term perspective (20 years).

## Abbreviations

BMD: bone mineral density; DXR: digital X-ray radiogrammetry; DXR-BMD: bone mineral density measured by digital X-ray radiogrammetry; RA: rheumatoid arthritis.

## Competing interests

JA is employed by Sectra but is not a shareholder in the company. All other authors have declared no competing interests. The radiographs were digitized using a Vidar Diagnostic Pro Plus digitizer (VIDAR Systems Corp., Herndon, VA, USA), and the resulting digital radiographs were analyzed using DXR online (Sectra, Linköping, Sweden), but any financial or other support was obtained from the company.

## Authors' contributions

All authors have made substantial contributions to the study's conception and design, acquisition of data or analysis and interpretation of data and have been involved in drafting the manuscript or revising it critically for important intellectual content. MCK, EL and PG wrote the manuscript. EL and PG helped plan the study. JA developed the DXR method. KJL scored the radiograms. EL, TS and KE conceived the study. MCK helped in performing the statistical analysis. KE conceived the original Lund early RA cohort study. PG handled the database. All authors read and approved the final manuscript.
